# Data-Driven Prediction for COVID-19 Severity in Hospitalized Patients

**DOI:** 10.3390/ijerph19052958

**Published:** 2022-03-03

**Authors:** Abdulrahman A. Alrajhi, Osama A. Alswailem, Ghassan Wali, Khalid Alnafee, Sarah AlGhamdi, Jhan Alarifi, Sarab AlMuhaideb, Hisham ElMoaqet, Ahmad AbuSalah

**Affiliations:** 1 Department of Medicine, King Faisal Specialist Hospital & Research Centre, Riyadh 11211, Saudi Arabia; 2 Healthcare Information & Technology Affairs, King Faisal Specialist Hospital & Research Centre, Riyadh 11211, Saudi Arabia; 3 Department of Medicine, King Faisal Specialist Hospital & Research Centre, Jeddah 21561, Saudi Arabia; gwali@kfshrc.edu.sa; 4 Infection Control & Hospital Epidemiology Department, King Faisal Specialist Hospital & Research Centre, Riyadh 11211, Saudi Arabia; kalnafee@kfshrc.edu.sa; 5 Center of Healthcare Intelligence, Health Information & Technology Affairs, King Faisal Specialist Hospital & Research Centre, Riyadh 11211, Saudi Arabia; salghamdi2@kfshrc.edu.sa (S.A.); jalarifi@kfshrc.edu.sa (J.A.); abusalah@kfshrc.edu.sa (A.A.); 6 Computer Science Department, College of Computer & Information Sciences, King Saud University, Riyadh 11451, Saudi Arabia; almuhaideb@ksu.edu.sa; 7 Department of Mechatronics Engineering, German Jordanian University, Amman 11180, Jordan

**Keywords:** COVID-19, severity prediction, decision support systems, applied artificial intelligence, hospital operations

## Abstract

Clinicians urgently need reliable and stable tools to predict the severity of COVID-19 infection for hospitalized patients to enhance the utilization of hospital resources and supplies. Published COVID-19 related guidelines are frequently being updated, which impacts its utilization as a stable go-to resource for informing clinical and operational decision-making processes. In addition, many COVID-19 patient-level severity prediction tools that were developed during the early stages of the pandemic failed to perform well in the hospital setting due to many challenges including data availability, model generalization, and clinical validation. This study describes the experience of a large tertiary hospital system network in the Middle East in developing a real-time severity prediction tool that can assist clinicians in matching patients with appropriate levels of needed care for better management of limited health care resources during COVID-19 surges. It also provides a new perspective for predicting patients’ COVID-19 severity levels at the time of hospital admission using comprehensive data collected during the first year of the pandemic in the hospital. Unlike many previous studies for a similar population in the region, this study evaluated 4 machine learning models using a large training data set of 1386 patients collected between March 2020 and April 2021. The study uses comprehensive COVID-19 patient-level clinical data from the hospital electronic medical records (EMR), vital sign monitoring devices, and Polymerase Chain Reaction (PCR) machines. The data were collected, prepared, and leveraged by a panel of clinical and data experts to develop a multi-class data-driven framework to predict severity levels for COVID-19 infections at admission time. Finally, this study provides results from a prospective validation test conducted by clinical experts in the hospital. The proposed prediction framework shows excellent performance in concurrent validation ( n=462 patients, March 2020–April 2021) with highest discrimination obtained with the random forest classification model, achieving a macro- and micro-average area under receiver operating characteristics curve (AUC) of 0.83 and 0.87, respectively. The prospective validation conducted by clinical experts ( n=185 patients, April–May 2021) showed a promising overall prediction performance with a recall of 78.4–90.0% and a precision of 75.0–97.8% for different severity classes.

## 1. Introduction

The Coronavirus Disease 2019 (COVID-19) pandemic has presented unprecedented challenges and threats for health care systems worldwide [1,2,3,4,5,6]. Since the initial outbreak in early December 2019, the number of patients reported to have the disease has exceeded 395 million in more than 160 countries, and the number of people infected is probably much higher. As the end of January 2022, more than 5 million people have died from COVID-19 [7].

Despite public health responses aimed at containing the disease and delaying its spread, several countries have faced a critical care crisis, and more countries will almost certainly follow [8,9,10]. As of February 2022, more than 10 billion doses of various vaccines have been administered globally [11]. Nevertheless, countries worldwide are still experiencing surges in the number of COVID-19 cases, as well as successive waves of the pandemic resulting from the virus and its continuously arising variants, in spite of aggressive vaccination efforts [12,13]. Outbreaks lead to important increases in the demand for hospital beds and shortage of medical equipment, and medical staff themselves are at high risk of infection.

To alleviate the burden on health care systems, while providing the best possible care for patients, efficient clinical diagnosis and prognosis of COVID-19 is of significant importance. Recent studies on COVID-19 have proposed many statistical models that can combine several variables or features to estimate the risk of infection or experiencing a bad outcome from the infection. These models can be grouped into three main categories: models to indicate the disease risk in the general population, diagnostic models to detect COVID-19 in patients with suspected infection, or prognostic models for patients with diagnosis of COVID-19 [14]. Models ranging from rule-based scoring systems to deep learning models have been proposed and published [14].

Several studies for large observational cohorts of COVID-19 patients have reported clinical characteristics that are associated with severe illness and death. However, these findings are difficult to translate to the quantification of absolute risk in the context of informing care at the level of individual patients [1,3,15,16,17]. Many clinical risk calculators have been created [14,18,19,20] during the early stages of the pandemic. Nevertheless, there are currently no reliable patient-level prediction tools in widespread use for COVID-19 due to many limitations in the existing prediction models [21]. These limitations include disregarding important clinical information upon hospital admission as well as problems related to model generalization and clinical validation [14,22].

A major limitation in previous prognosis studies is that they were primarily conducted on severe forms of the disease [22]. Research on asymptomatic, mild, or moderate COVID-19 infections is limited [23,24]. From a practical perspective, and considering limited clinical assessments on the hospital floor, triage decisions are mostly complicated by COVID-19’s biphasic clinical course: patients who present initially with mild symptoms often return later for admission, and many subsequently suffer adverse events, including ICU transfer, mechanical ventilation, or mortality [25]. Thus, developing reliable methods to predict COVID-19 infection severity levels will provide significant assistance for clinicians in appropriately triaging and planning follow-up care for patients presenting for COVID-19 screening, facilitating improved allocation of limited health care resources.

In this paper, we develop and deploy a novel data-driven prognosis framework to provide patient-level predictions for the severity of COVID-19 infection for patients at the time of hospital admission. To overcome the limitations of current approaches and respond to the continuous clinical demand for robust and individualized prediction of COVID-19 severity, we leverage a well-curated data registry of patients admitted with COVID-19 to one of the largest tertiary hospitals in the Middle East. Unlike many previous studies that consider a single prediction model, in this study, we comprehensively evaluate and compare four machine learning models. Moreover, the proposed prediction framework was evaluated using concurrent and prospective validation data sets in order to ensure the results’ generalizability.

## 2. Materials and Methods

### 2.1. Data Sources

King Faisal Specialist Hospital and Research Centre (KFSH&RC) is located in the Kingdom of Saudi Arabia (KSA) and is among the largest tertiary multi-hospital systems and medical research centers in the Middle East region. Its main facilities are located in Riyadh (the capital of KSA), Jeddah, and Madinah. Combined, the hospital has more than 1800 beds and 14,000 staff and clinical consultants. On average, it has 30,000 admissions and 90,000 emergency room (ER) visits each year [26]. The Automated Multi-Dimensional COVID-19 Registry System is a well curated registry system developed at KFSH&RC to support research, operations, and health care intelligence. This data registry system was used as the primary data source for those patients hospitalized with COVID-19 considered in this study.

Our study cohort included two groups of subjects. The first group contained 1848 adult patients admitted to the hospital between March 2020 and 6 April 2021. Of these, 49.32% were male and the remaining 50.68% were female patients. Adult patients in this study included only those who were of age ≥18 years at the time of their COVID-19 related hospital admission. Using stratified sampling, this data set was split as follows: 75% (1386 patients) was used for training and model development and 25% (462 patients) was used for testing and model evaluation (concurrent validation). Patient data in the second group were considered as a prospective validation set as the data were collected after the end of the collection period for the original data set. Subjects in the second group included 185 adult COVID-19 patients admitted to the hospital between late April and May 2021.

**Figure 1:**
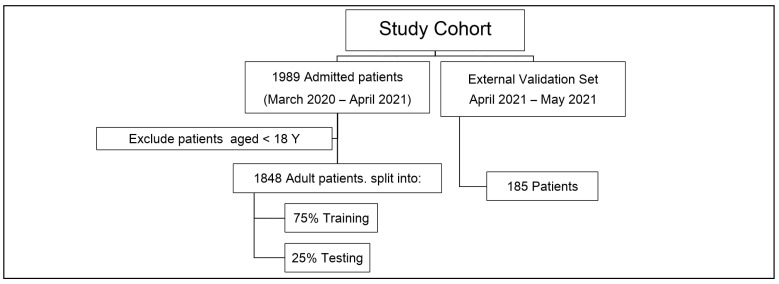
Details of the study cohort and data sets used for model development, testing, and validation.

The recorded data for each patient in both groups encompass clinical observations collected from inpatient encounters. Patients’ race and ethnicity were excluded from the study. In addition, all members of the patient population are from the same hospital. Figure 1 shows the details of the study cohort and the data sets used for model development, evaluation, and prospective validation.

COVID-19 hospital admission terms for subjects in this study included: general admission, ICU admission, anesthesia and critical care medicine admission, chronic care management admission, and critical care admission. For patients with multiple admissions, we considered the earliest date and time of admission. Patients admitted with COVID-19 were identified using the following severity stages: *A* = asymptomatic, *B* = mild, *C* = moderate, and *D* = severe according to the guidelines of the U.S. National Institutes of Health (NIH) [27], which are used in the KSA.

### 2.2. Features

For each patient, the following features were considered:

(1) **Demographic features** included the blood type, age, and gender of the patients admitted with COVID-19. In addition, height and weight were used to compute the Body Mass Index (BMI) as a demographic feature.
(2) **Vital signs** included body temperature (axillary and oral temperature), heart rate, and oxygen saturation at admission. Maximum heart rate, body temperature, and respiratory rate, as well as the minimum oxygen for a 24-h interval after admission, were recorded and considered.
(3) **PCR cycle threshold (Ct) value** was used to categorize patients in terms of the viral load associated with the COVID-19 infection. Ct ≥ 34 was categorized as low viral load, 26 ≤ Ct < 34 was categorized as medium viral load, and Ct < 26 was categorized as high viral load. The Ct value taken was the closest before time of admission.
(4) **Health conditions** of patients admitted with COVID-19 included 12 such conditions that were used to describe the admitted patients. These conditions included information about specific chronic diseases and major treatments. Chronic diseases considered included diabetes mellitus, hypertension, cancer, heart failure, coronary arterial disease, chronic lung and kidney diseases, and human immunodeficiency virus (HIV) infection. Furthermore, information about patients with obesity, chronic use of steroids, solid organ stem cell transplant, and treatment with chemotherapy for cancer was also considered. Each of these health conditions was evaluated independently, using a flag of Yes/No to represent the presence/absence for each of them, respectively.
(5) **Severe acute respiratory infection (SARI) risk exposure** which included two risk levels. The first, directly exposed persons, included persons who were within two meters of an asymptomatic patient without the use of an N95 respiratory mask and without considering droplet and contact precautions. The other type of exposure risk is denoted by non-directly exposed persons, including those who stayed in the same environment with an asymptomatic patient before implementing airborne transmission precautions while implementing droplet and contact precautions. Based on this criterion, patients were categorized into directly exposed (34%), indirectly exposed (16%), and unknown (46%) groups. Unlike other features which were recorded by the EMR system, this feature is patient reported data.

### 2.3. Data Preprocessing and Preparation

The collected data set included some missing feature values that represent unknown values for these features in corresponding patients. Missing measurements is a very common issue in medical data registries. Our analysis showed no systematic differences between patients with missing data and those with complete data. Thus, missing data in this study were assumed to be missing completely at random (MCAR) [28]. In this study, missing values were imputed using a pipeline based on the K-nearest neighbors (KNN) approach. For any missing patient feature, we identified the patient with features that were closest in value to the features of the patient with the missing feature (i.e., the patient with the closest health state). Then, we used model-based imputation methods to generate estimates for the missing parameters conditional on the given data that we had, the observed relationship between variables, and constraints imposed by the underlying distributions. Different modeling methods were evaluated and the method that provided the minimum cross validation mean square error (MSE) over training data was selected for imputation. Thus, each missing value was estimated using various regression models (i.e., random forest (RF), Bayesian ridge, Extra Trees) by calculating the mean squared error (MSE) and selecting the estimation method with least MSE for imputation.

After that, the data were prepared for use with machine learning algorithms. The categorical feature variables were represented using the one-hot encoding approach, whereas the numeric continuous feature variables were standardized by subtracting the mean value from each of them so that the standardized values had a zero mean, and then by dividing the result by the standard deviation ( σ ) so that the resulting distribution had unit variance.

Standardizing the features also enabled us to obtain a *Z*-score (with zero mean and unit variance), which can be used for outlier detection and removal using standard *Z*-score cut-off values. For each scaled feature, outliers were identified by a *Z*-score cut-off =5σ .

**Figure 2:**
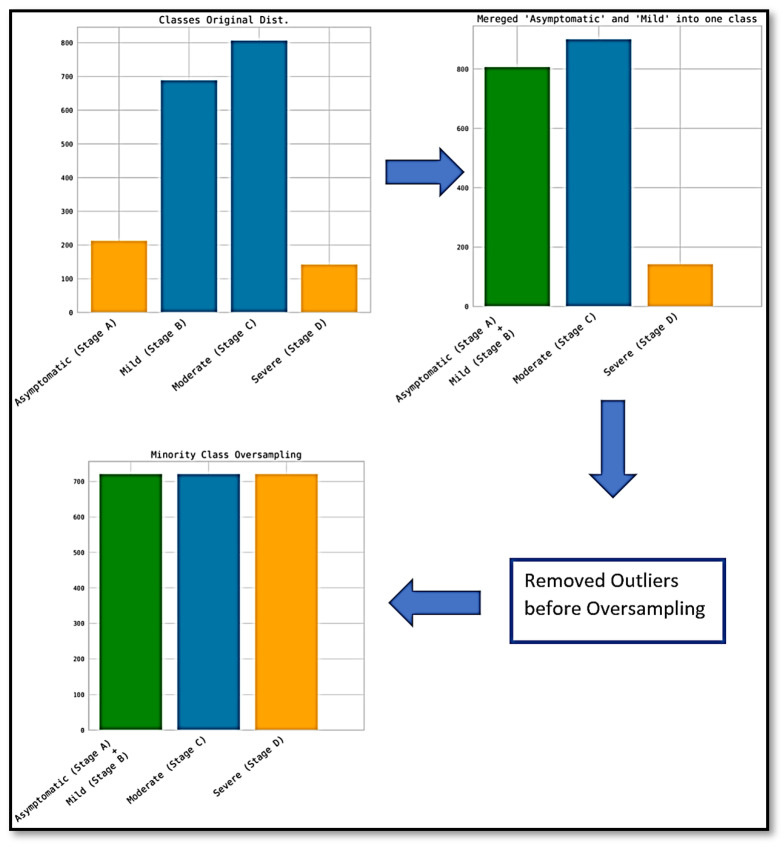
Distribution of training data after merging classes A and B and oversampling minority classes.

The distribution of patients in the modeling data set included 13.1% asymptomatic, 38.2% mild, 41.6% moderate, and 7.2% severe patients. Two actions were taken to overcome imbalance between the classes and avoid introducing bias into the prediction results due to this imbalance. First, and upon discussing the matter with clinicians, we merged asymptomatic (*A*) and mild (*B*) classes into one class, AB , due to their similarity. Then, we oversampled the minority classes (classes C and D) so that all classes had equivalent distribution (representation) within the training set. We intentionally removed outliers prior to the oversampling process so that the outliers would not be emphasized in the generated oversampled distribution. This is further illustrated in Figure 2.

### 2.4. Prediction Philosophy and Classification Models

In this study, we aimed to predict the severity of COVID-19 infections using readily available clinical data and physical examination findings at the time of admission to the hospital. Thus, we posed this problem as a multi-class prediction problem. For each patient *j* where j=1,2,…N , and *N* = 1848 is the number of patients in the study group, we can construct the data tuple { $x_{j},t_{j}$ } where $x_{j}$ denotes the *m*-dimensional feature vector for patient *j*, $x_{j}={\left[x_{1j},\ldots ,x_{mj}\right]}^{T}$ . This is composed of the features $x_{1j},\ldots ,x_{mj}$ , where m=27 is the total number of features extracted for each patient, $t_{j}$ is the target severity class label of the patient *j*, $t_{j}\in \left\{1,2,\ldots K\right\}$ where *K* is the number of severity classes considered in this study ( K=3 ). The goal is to identify a classification model that can predict the severity class label $t_{j}\in \left\{1,2,\ldots ,K\right\}$ using the corresponding feature vector $x_{j}$ , for each patient j∈N . To identify the most feasible model and ensure the generalizability of the proposed framework, we evaluated four different machine learning models that support multi-class predictions including multinomial logistic regression (a classical classification model well known in the medical field) and three ensemble based learning algorithms.

#### 2.4.1. Multinomial Logistic Regression (MLR)

MLR generalizes traditional LR to multiclass problem [29]. MLR implements a linear predictor function $f\left(x_{j},k\right)=\omega^{\left(k\right)}x_{j}$ , where f(·) obtains the score of $x_{j}$ belonging to class *k*, depending on the vector of logistic regressors corresponding to the *k*th class, defined as $\omega^{\left(k\right)}=\left[\omega_{0}^{\left(k\right)},\omega_{1}^{\left(k\right)},\ldots ,\omega_{m}^{\left(k\right)}\right]$ . MLR is formally defined by the following probability density function (PDF) [30]

(1) $$p\left(t_{j}=k|x_{j};\Omega \right)=\frac{exp(\omega^{\left(k\right)}x_{j})}{\Sigma_{k=1}^{K}exp\left(\omega^{\left(k\right)}x_{j}\right)}$$

where $\Omega =[\omega^{\left(1\right)};\ldots ;\omega^{\left(K\right)}]$ comprises the logistic regressors of all classes. Indeed, the main goal of the MLR is to estimate the Ω set from the training data set.

Considering the MLR as a set of independent binary regressions, the method runs K−1 models and selects one label (normally the *K*th class) as the “pivot” and separately regress the remaining K−1 labels against the pivot by the following equation:

(2) $$\frac{p(t_{j}=k|x_{j})}{p(t_{j}=K|x_{j})}=exp\left(\omega^{\left(k\right)}x_{j}\right)$$

where the index k∈{1,2,…,K−1} has been corrected and *K* is the pivot label. The MLR is interpreted as a set of K−1 independent logistic regression models for the probability of $t_{j}=k$ versus the probability of the pivot label $t_{j}=K$ . Recognizing that all class probabilities must sum to one $\Sigma_{k=1}^{K}p\left(t_{j}=k\right)=1$ , we can generalize Equation (2) to express the pivot class probability as follows:

(3) $$p\left(t_{j}=K|x_{j}\right)=\frac{1}{1+\Sigma_{k=1}^{K}exp\left(\omega^{\left(k\right)}x_{j}\right)}$$

Paragraph format $\omega^{\left(k\right)}\in \Omega$ are calculated using a regularized maximum likelihood estimation. Regularization is controlled by a hyperparameter that is tuned to reject complex models and improve generalization of the estimated MLR model over unseen data. The final solution for the best estimate of the unknown model parameters can be found using iterative search procedures, such as gradient-based optimization algorithms [31].

#### 2.4.2. Random Forest (RF)

RF is an ensemble machine learning method that constructs a multitude of decision trees at training time and outputs the class that is averaged or voted by every individual tree [32]. RF was proposed by Breiman in 2001, who added an additional layer of randomness to the bagging method [32].

Bagging, or bootstrap aggregating, is an ensemble algorithm designed to improve the stability and accuracy of individual predictive models such as trees [33]. Bagging helps decision trees reduce their variance, making RF one of the most popular ensemble tree models, mainly due to its stability and robustness with data sets of any size. When training an RF, individual decision trees are trained using different samples of the instances (bagging method), whereas at each split, the learning algorithm randomly samples a subset of the features and chooses the best split among them. Finally, multiple base classifiers are combined into an RF model, and the final model classification result is obtained by majority voting.

Previous theoretical and experimental studies have shown that the RF has high prediction accuracy and generalization performance [34]. It has good robustness for noisy data and missing values. Most importantly, it does not suffer from overfitting because it uses the predictions’ average of all decision trees participating in the classification or regression process, canceling out the biases. Model hyperparameters that were tuned for the RF include the maximum allowable depth per tree, maximum number of features per node split, and the number of estimators (trees) in the forest.

#### 2.4.3. Extreme Gradient Boosting (XGBoost)

XGBoost is a scalable machine learning ensemble system for tree boosting proposed in 2016 [35]. Gradient boosting is the original XGBoost model; it improves weak classifier models sequentially and makes them strong classifiers [36]. The idea behind this algorithm is to construct multiple decision trees based on feature splitting nodes. As a decision tree-based model, XGBoost learns sequentially from residuals using the residual fitting approach to increase the accuracy of data classification. Each time a decision tree is constructed, the residual predicted by the last model is fitted, so that the objective function is reduced (i.e., performance is improved). In other words, gradient boosting technique sequentially generates a new model to predict the residual of previous tree models and gradually increases performance thereby [35]. Finally, many weak decision tree classifiers are integrated into a strong classifier, and each leaf node of each tree corresponds to a score. When a sample is predicted, the model will find the corresponding leaf nodes in each tree based on the characteristics of the sample. The predicted value of the sample is the sum of the score of all leaf nodes. Model hyperparameters that were tuned for the XGBoost include the learning rate, maximum depth per tree, and the number of estimators (trees).

#### 2.4.4. Extremely Randomized Trees (Extra Trees)

The extremely randomized trees (Extra Trees) classifier is an ensemble learning method that was proposed in 2006 [37]. Extra Trees build multiple decision trees in a forest and aggregate their votes to output the classification result. In this method, the nodes are split using random subsets of features rather than best splits. The Extra Trees classifier differs from the RF classifier in constructing the decision trees in the forest and selecting the split point. By comparing the execution time and computational cost, the Extra Trees algorithm is faster than the RF algorithm because it selects the optimal split randomly instead of searching for it at each node. Model hyperparameters tuned for Extra Trees include the maximum depth per tree, minimum number of training samples per node split, and the number of estimators (trees).

### 2.5. Evaluation of Prediction Results

In this study, recall ( Re ), precision ( Pr ), and $F_{1}$ score were applied to assess the performance of the proposed modeling framework in predicting each of the severity classes k∈{1,2,…,K} . Re is the fraction of patients who were correctly predicted to fall into severity class *k* out of all patients with target class label *k*. Pr is the fraction of patients who were correctly predicted to fall into severity class *k* out of all patients who were predicted for this severity class. The $F_{1}$ score is the harmonic mean of Re and Pr and can account for the Re / Pr tradeoff, providing a more comprehensive snapshot of the overall performance of the proposed modeling framework.

Additionally, the receiver operating characteristics ( ROC ) curve was used to illustrate the diagnostic ability to predict each severity class [38]. The area under receiver operating characteristics curve ( AUC ) was used as a measure of the classification’s model overall ability to predict different severity classes. A greater AUC indicates a more useful and effective classification model.

## 3. Experiments and Results

### 3.1. Classification Performance over Test Set of Patients

The training data set was used for model development. To tune the hyperparameters for each model, we performed a grid search with a five-fold cross-validation using the training data set. We repeated this 10 times to ensure optimality of the hyperparameter selections. Table 1 shows the optimal selection for each of the tuned hyperparameters per each model. The testing data set was isolated and used only to evaluate and compare the performance of the developed models.

**Figure 3:**
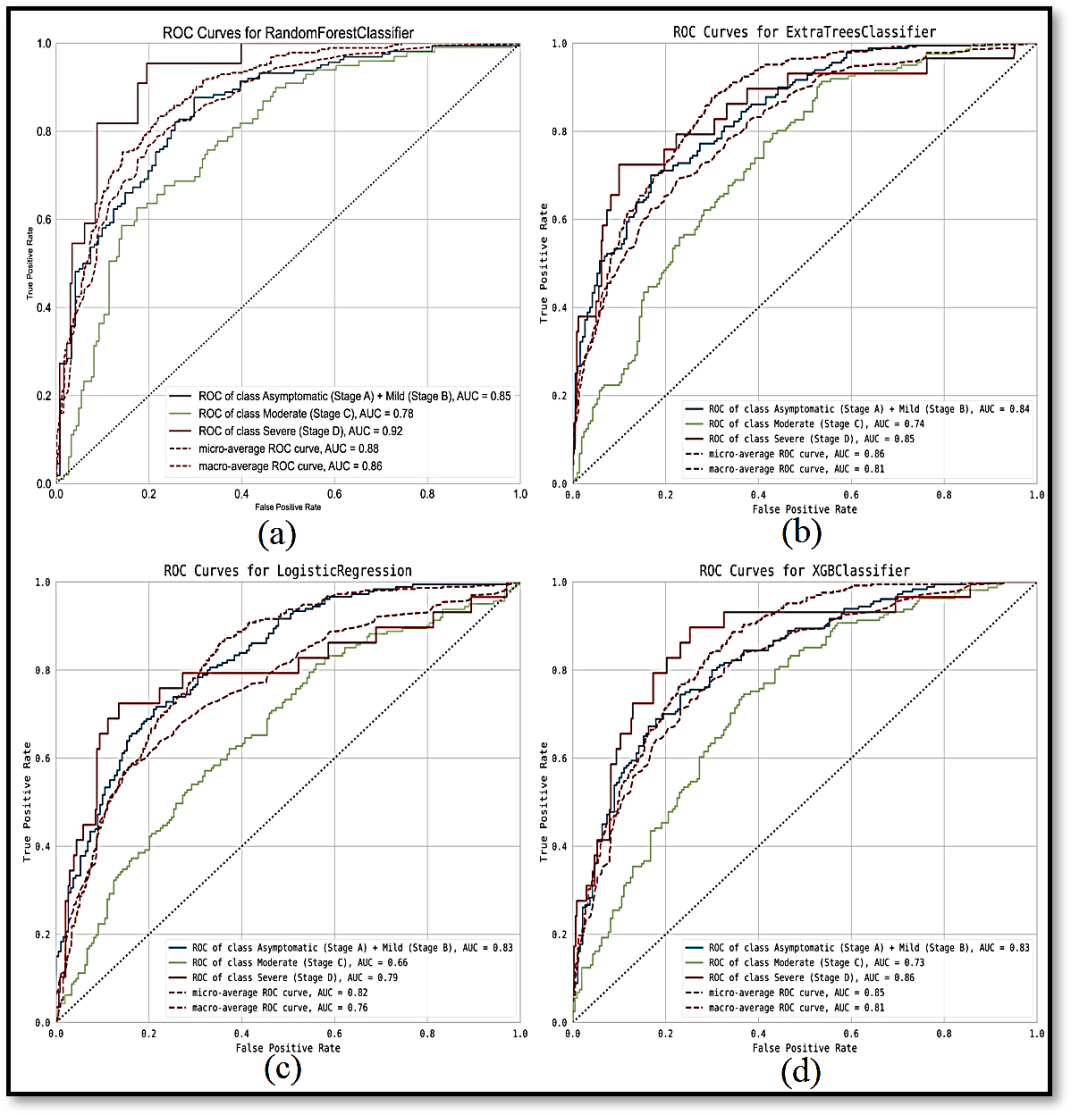
ROC for different classification models over test patient data: (**a**) RF classifier, (**b**) Extra Trees classifier, (**c**) multinomial logistic regression, (**d**) XGB classifier.

Figure 3 and Table 2 show test ROC results and the corresponding AUC performance for the classification models employed in this study. The ensemble methods show a clearly improved AUC performance over the MLR method. Among the three tree-based classification models, the RF classifier shows the best classification results (for three severity classes) as well as the respective micro- and macro-averages for the AUC values. Thus, the RF classifier was selected to implement and deploy the severity prediction system at KFSH&RC.

### 3.2. Implementation and Evaluation over Prospective Validation Data

The proposed system was implemented and further developed into an intelligent software engine for COVID-19 patient severity level assessment and prediction at the time of admission to the KFSH&RC. Furthermore, the proposed prediction framework was prospectively validated on a data set of 185 patients as shown in Figure 1.

Table 3 shows the per-class performance of the proposed COVID-19 severity prediction system in predicting the severity of 185 COVID-19 admissions in the period from late April to May 2021. As the table shows, the proposed prediction tool shows a very good overall performance in predicting COVID-19 severity levels at admission, providing excellent assistance to clinicians and the clinical decision-making process regarding treatment and resource allocation plans for newly admitted patients. Interestingly, the highest recall ( Re ) values were achieved during the combined Stage AB , which indicates the model has an excellent ability to rule out those who do not need close monitoring and treatment. Accordingly, this will improve resource allocation and utilization for those who are in urgent need of close health care supervision. Furthermore, the model has superior Pr values in predicting severe COVID-19 cases, which will also have a significant impact on clinical decisions to directly prepare those assessed as being in the severe class for advanced treatment protocols.

### 3.3. Feature Importance in Predicting COVID-19 Severity

Overall, the best preforming model for prediction in this study was the RF classifier. This model is not only applicable in regression and classification but also has excellent behavior in feature ranking and selection. In other words, RF can be used to sort features with respect to their contribution in predicting the target COVID-19 severity classes [39].

Despite providing sensible means for feature selection for many applications, RF feature importance may be unreliable in situations where features vary in their scale of measurement or number of categories, which is very common in biomedical applications [40]. Thus, for model inspection purposes, we considered the standard RF feature importance analysis, as well as an alternative RF implementation, that provides unbiased variable selection and ranking in the individual classification trees which is called the permutation feature importance method [40]. It is a model inspection technique that can be used for any fitted estimator when the data are structured or tabular, and it is especially useful for non-linear and opaque estimators [40]. The permutation feature’s importance is defined to be the decrease in a model score when a single feature value is shuffled randomly [32]. This procedure breaks the relationship between the feature and the target, so a decrease in the model score indicates how much the model depends on the feature. This technique is model agnostic and can be calculated many times with different permutations of the feature. In this study, we considered permutation feature’s importance using RF as a base estimator since this was the best performing model among those considered earlier.

**Figure 4:**
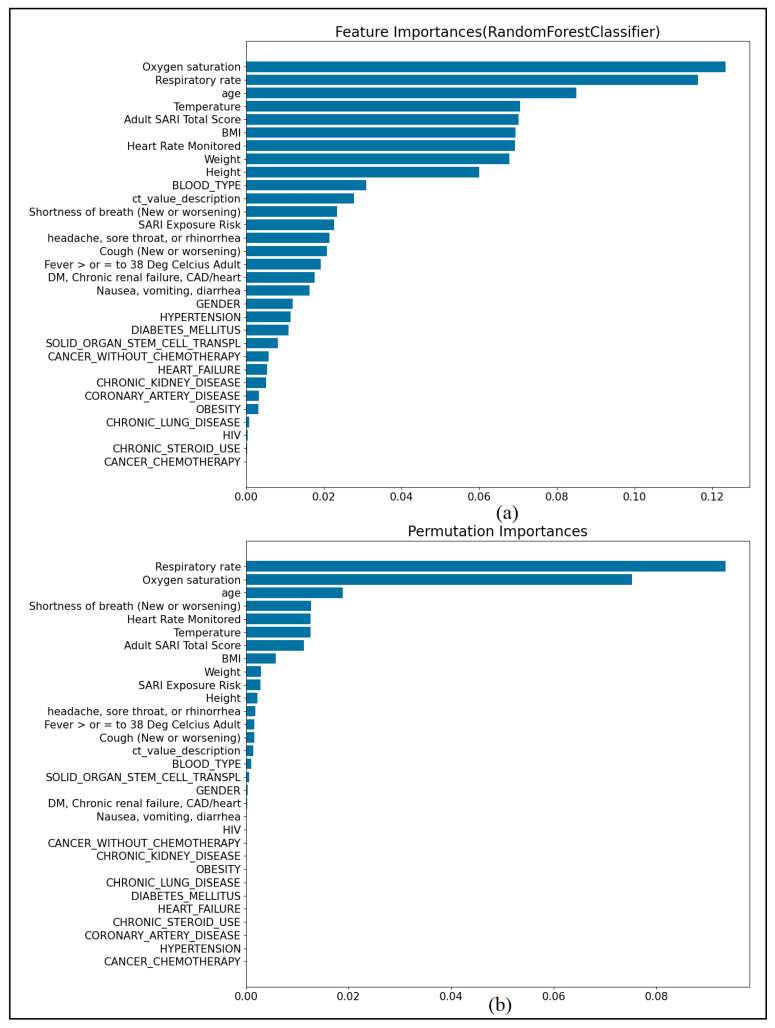
Feature importance in predicting COVID-19 severity: (**a**) standard RF feature importance and (**b**) permutation feature importance.

Figure 4 shows the feature importance ranking for predicting the severity of COVID-19 infection using the standard RF importance method and with permutation. It is clear that the respiratory rate and the blood oxygen saturation levels are the most important factors in predicting the severity of COVID-19 infection for patients admitted to the hospital. Despite the differences in ranking between the two methods, both give high importance for temperature, heart rate, age, and BMI.

## 4. Discussion

In this study, we rigorously developed and prospectively validated a data-driven framework to predict the severity of COVID-19 infection in patients admitted to the hospital. The prediction model uses readily available clinical data and physical examination findings at the time of admission to the hospital as inputs to the prediction framework. Unlike previous studies that considered only one prediction model, our study comprehensively evaluated and compared four machine learning models including an MLR model and three tree-based ensemble learning models (XGBoost, Extra Trees, and RF). Our results indicate that the RF model obtained the best prediction performance. Data from 1386 patients were used for model training and optimization. The proposed prediction system shows excellent discrimination results in both concurrent validation ( n=462 , March 2020–6 April 2021) and prospective validation ( n=185 , late April–May 2021).

Several previous studies developed prognosis models for COVID-19 [25,41,42,43,44,45,46,47,48,49,50,51,52]. Nevertheless, these models were limited to predicting severe illness [41,42,44,52], mortality [45,46,47,48,49,50,53], or (less frequently) the length of stay in hospital for COVID-19 patients [45,46,47,48,49,50]. The majority of these studies considered various patient lab measurements as features for prediction [41,43,45,46,48,52,53], while others considered features from lung tomography/radiography images [25,41,43,46,51,52]. Not only that the majority of these models were limited to predicting adverse conditions but they were also based on small patient cohorts (median, *n* = 189 [25]), developed for the inpatient and not accounting for outpatient screening, and have rarely been prospectively validated [44,47,51,54].

The present study is distinct from previous studies in many aspects. First, we use a large data set of COVID-19 patients with diverse severity stages ranging from asymptomatic to severe. This data set was leveraged by a panel of clinical and data experts to develop a multi-class data-driven framework that is optimized to predict different COVID-19 severity levels and not only limited to critical illness and death. Second, our study uses COVID-19 patient-level clinical data that is easily accessible and commonly available at the hospital floor. The proposed approach eliminated the need to utilize complex laboratory values that would limit the applicability of the model to specific lab input values which might not be readily available. More importantly, the performance of the proposed framework is not dependent on adjustable cut-off thresholds applied on lab measurements which is a significant limiting factor as thresholds might not be generalizable to different patient populations [22]. Finally, we validated our model on a prospectively collected patient cohort, providing an assessment of model generalizability with reduced (minimal) bias.

Our analysis shows that simple data extracted from EMR, vital signs, and PCR machines are of significant importance in predicting the severity of COVID-19 infection at the time of hospital admission. In particular, our results show that respiratory rate and blood oxygen saturation levels are the most significant predictors for the severity of a COVID-19 infection. Interestingly, this agrees with previous research findings [17] despite differences in patient demographics and data analysis methods. Other variables that were found to significantly contribute to the disease severity include advanced age, heart rate, temperature, and obesity (high BMI), which also have been identified as significant risk factors in some recent studies [3,4,25,55,56,57]. Similar to [25], our results indicate that hypertension and diabetes mellitus are not significant predictors for COVID-19 severity.

The proposed system showed promising prediction performance in both concurrent and prospective validation. Furthermore, it has been further developed to an intelligent software engine for COVID-19 patient severity level assessment and prediction at the time of admission to KFSH&RC. Nevertheless, and similar to other clinical decision support systems, it does not totally replace physician judgment. Rather, it will significantly help to improve risk stratification for COVID-19 patients and enhance medical decisions regarding testing, hospitalization, and follow-up. The proposed prediction framework will also assist providers in matching patients with appropriate level of needed care for better management of scarce hospital resources during COVID-19 surges.

Our study has some limitations. Although the study is based on a data registry system from a large tertiary hospital system, it is still a single health care system. Consequently, this may limit the generalizability of the proposed severity prediction tool to other health care systems. Another limitation of this study is that it did not consider patients who were not admitted to the hospital. As a result, we may not have captured hospitalizations and deaths that occurred outside KFSH&RC. Nevertheless, we believe most patients would have been readmitted to KFSH&RC and the electronic medical records (EMR) would have captured details of their clinical status.

Future efforts will focus on incorporating more data from other healthcare systems into the prediction system in order to extend its use to regional and national levels. Additionally, we plan to further improve the performance of the prediction framework using features from the respiration and blood oxygenation waveforms. We plan to use the dynamic changes in breath amplitudes and inter-breath intervals as well as the frequency and depth of oxygen desaturations [58,59,60] in order to improve the overall performance of the prediction framework and particularly improve the ability to predict the class of moderate COVID-19 infections.

## 5. Conclusions and Future Work

Despite more than 10 billion doses of different types of vaccines that have been administered globally, the world still experiences continuous surges in the number of COVID-19 cases resulting from the virus and its continuously arising variants. This paper provides a new perspective for predicting patients’ COVID-19 severity levels at the time of hospital admission using comprehensive data collected from the hospital electronic medical records (EMR), vital sign monitoring devices, and Polymerase Chain Reaction (PCR) machines. Our study comprehensively evaluates and compares 4 machine learning models using a training data set of 1386 patients (March 2020–April 2021). Data-driven models that were investigated included multinomial logistic regression (MLR) and three tree-based ensemble learning models (XGBoost, Extra Trees, and RF). Our results indicate that the best prediction performance was obtained with the RF model. Moreover, experiments showed excellent discrimination results in concurrent validation ( n=462 patients, March 2020–April 2021) and prospective validation ( n=185 patients, April–May 2021). The proposed framework was developed into a real-time severity prediction tool in KFSH&RC which significantly assisted in matching patients with appropriate level of needed care during COVID-19 surges.

Furthermore, our results show that respiratory rate and blood oxygen saturation levels are among the most significant predictors for the severity of COVID-19 infections. Future work may focus on extending the framework to analyze continuous waveforms of respiration and blood oxygenation to improve the prediction performance as well as integrating patient data from several health care centers in order to extend the use of this prediction framework into a large scale.

## Figures and Tables

**Table 1:** Optimal hyperparameter settings for the different machine learning models considered in this study.

Model	Hyperparameter	Best Selection
MLR	Reg. penalty	L2 Norm
	Reg. Coeff.	1
RF	max_depth	60
	max_features	Auto
	n_estimators	800
XGBoost	learning_rate	0.1
	max_depth	9
	n_estimators	100
Extra Trees	max_depth	100
	n_estimators	500
	min_samples_split	5

**Table 2:** AUC Results for different classification models using test patient data (concurrent validation).

AUC / ROC Results Using Test Patient Data					
**Model**	**Stage** AB	**Stage** C	**Stage** D	**Micro-avg.**	**Macro-avg.**
MLR	0.83	0.66	0.78	0.82	0.76
XGBoost	0.82	0.72	0.88	0.85	0.81
Extra Trees	0.84	0.73	0.85	0.86	0.81
RF	0.86	0.75	0.88	0.87	0.83

**Table 3:** Severity prediction performance over prospective validation data.

RF Classification Performance over Validation Set			
**Severity Stage**	Re	Pr	$F_{1}$ **-Score**
Stage *A* (Asymptomatic) + Stage *B* (Mild)	90%	75.0%	81.8%
Stage *C* (Moderate)	78.4%	69.0%	73.4%
Stage *D* (Severe)	78.9%	97.8%	87.4%

## Data Availability

The data that support the findings of this study are available from King Faisal Specialist Hospital & Research Centre (KFSH&RC), but restrictions apply to the availability of these data. The data sets in this research were used under license for the current study, and so are not publicly available. Data sets of this study can be made available upon approval of a research request by the COVID-19 Command and Control Committee at KFSH&RC.
